# Effects of the catastrophic 2020 Yangtze River seasonal floods on microcystins and environmental conditions in Three Gorges Reservoir Area, China

**DOI:** 10.3389/fmicb.2024.1380668

**Published:** 2024-03-06

**Authors:** Yuanhang Zhou, Qilong Wang, Guosheng Xiao, Zhi Zhang

**Affiliations:** ^1^Key Laboratory of the Three Gorges Reservoir Regions Eco-Environment of Ministry of Education, College of Environment and Ecology, Chongqing University, Chongqing, China; ^2^Engineering Technology Research Center of Characteristic Biological Resources in Northeast Chongqing, College of Biology and Food Engineering, Chongqing Three Gorges University, Wanzhou, Chongqing, China

**Keywords:** microcystins, cyanobacteria, environment conditions, seasonal floods, Three Gorges Reservoir Area

## Abstract

**Introduction:**

During July and August 2020, Three Gorges Reservoir Area (TGRA) suffered from catastrophic seasonal floods. Floods changed environmental conditions and caused increase in concentration of microcystins (MCs) which is a common and potent cyanotoxin. However, the effects and seasonal variations of MCs, cyanobacteria, and environmental conditions in TGRA after the 2020 Yangtze River extreme seasonal floods remain largely unclear, and relevant studies are lacking in the literature.

**Methods:**

A total of 12 representative sampling sites were selected to perform concentration measurement of relevant water quality objectives and MCs in the representative area of the TGRA. The sampling period was from July 2020 to October 2021, which included the flood period. Organic membrane filters were used to perform the DNA extraction and analyses of the 16S rRNA microbiome sequencing data.

**Results:**

Results showed the seasonal floods result in significant increases in the mean values of microcystin-RR (MCRR), microcystin-YR (MCYR), and microcystin-LR (MCLR) concentration and some water quality objectives (i.e., turbidity) in the hinterland of TGRA compared with that in non-flood periods (*p* < 0.05). The mean values of some water quality objectives (i.e., total nitrogen (TN), total phosphorus (TP), total dissolved phosphorus (TDP), and turbidity), MC concentration (i.e., MCRR, MCYR, and MCLR), and cyanobacteria abundance (i.e., *Cyanobium_PCC-6307* and *Planktothrix_NIVA-CYA_15*) displayed clear tendency of increasing in summer and autumn and decreasing in winter and spring in the hinterland of TGRA.

**Discussions:**

The results suggest that seasonal floods lead to changes in MC concentration and environmental conditions in the hinterland of TGRA. Moreover, the increase in temperature leads to changes in water quality objectives, which may cause water eutrophication. In turn, water eutrophication results in the increase in cyanobacteria abundance and MC concentration. In particular, the increased MC concentration may further contribute to adverse effects on human health.

## 1 Introduction

In recent years, China has frequently experienced seasonal floods. During July and August 2020, southern China suffered from five catastrophic seasonal floods, each named from Yangtze River No. 1 to No. 5 Flood, to pass through the Three Gorges Dam (Wei et al., 2020). Moreover, the Three Gorges Reservoir (TGR) Area (TGRA) plays an important role in flood control, power generation, navigation improvement, and regional development in China (Li et al., 2019, 2020). During the formation of No. 5 flood, the passing run-off water reached the historical maximum flood discharge of 75,000 m^3^·s^−1^. The entire Chongqing section of the Yangtze River exceeded the warning water level by a large margin since 1981 (Tan and Schultz, 2021; Tang et al., 2021). Floods bring large amounts of nutrients, sediments, and pollutants from the upstream areas and surface runoff (Mohamed et al., 2015; He et al., 2018). Meanwhile, floods also change environmental conditions (He et al., 2018; Pruett et al., 2021). That may lead to increase of cyanobacteria and microcystins (MCs).

Microcystin (MC) is a common and potent cyanotoxin, which can cause liver damage, tumor promotion, and even death (Guo et al., 2016; Zheng et al., 2017; Chatterjee and More, 2023). Furthermore, MC is mainly produced by some cyanobacteria genera, such as *Cyanobium, Planktothrix, Anabaena, Aphanizomenon*, and *Clindrospermopsi* (Chen et al., 2016; Brózman et al., 2020; Österholm et al., 2020; Peng et al., 2023, 2024). Cyanobacteria is a type of photosynthetic prokaryotes that could form algal bloom under favorable environmental conditions, such as high nutrient levels, high light intensity, and high temperature (Paerl and Paul, 2012). Among the MC family, microcystin-RR (MCRR), microcystin-YR (MCYR), and microcystin-LR (MCLR) have the widest distribution and highest concentration in natural waters (Ho et al., 2011; Beversdorf et al., 2018; Liu et al., 2019; Chernoff et al., 2020). Photocatalytic oxidation of MC revealed that MCLR had the strongest acute toxicity, whereas the toxicities of MCYR and MCRR are relatively lower than that of MCLR but still significant (Chernoff et al., 2021). Currently, most research focus on the impact of MCs on the environment and organisms (Huang et al., 2016; Scherer et al., 2017; Zhang et al., 2017; Krausfeldt et al., 2019; Li et al., 2022). Research on the effects of MC concentration and environmental conditions during floods is still lacking. Surprisingly, the effects of 2020 seasonal floods on MCs and environmental conditions in the TGRA remain largely unclear. Therefore, the effects and seasonal variation of MC concentration, cyanobacteria abundance, and environmental conditions in the TGRA after seasonal floods must be investigated.

**Figure 1:**
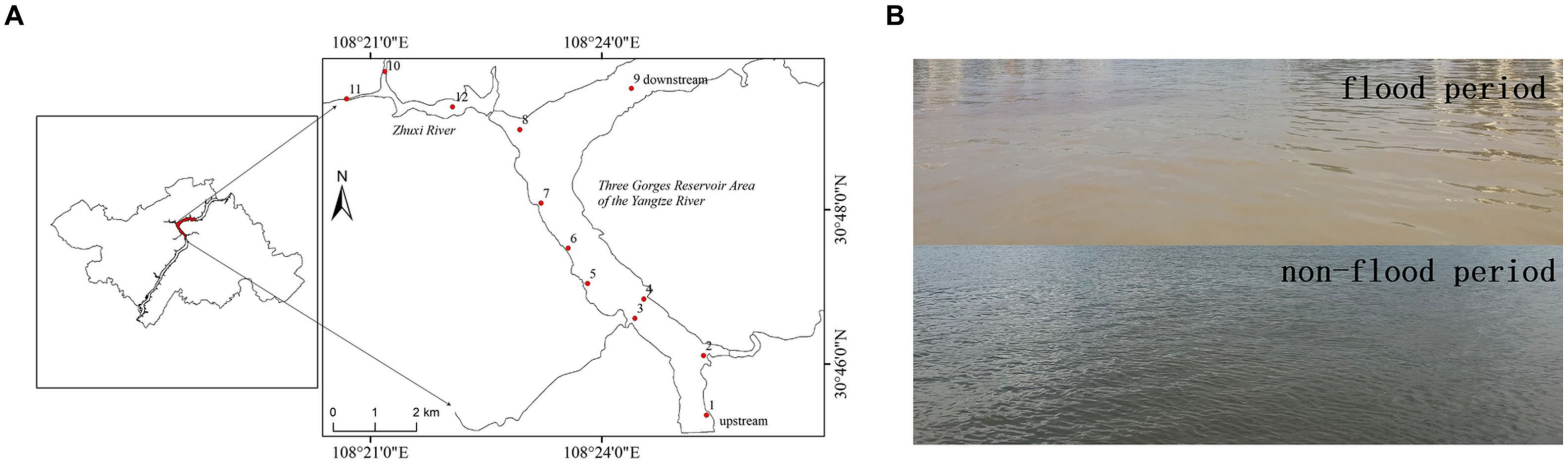
Map of the hinterland of the Three Gorges Reservoir Area (TGRA) of the Yangtze River showing the location of the 12 sampling sites **(A)**. Representative photograph of water quality during the flood (August 2020) and non-flood period (August 2021) **(B)**.

In this study, we performed a monthly sampling of 12 sites in the Wanzhou section within the hinterland of the TGRA of Yangtze River (Figure 1A) for 16 months, that is, from July 2020 to October 2021. Relevant water quality objectives and concentrations of MCs from the water samples were measured. Illumina high-throughput sequencing technology was also applied to analyze the abundance and composition of cyanobacteria in the water samples. The aims of this study are presented as follows: (1) evaluate the effects of seasonal floods on MC concentration, cyanobacteria abundance, and environmental conditions in the hinterland of the TGRA; (2) assess seasonal variation in MC concentration and environmental conditions in the hinterland of the TGRA; and (3) clarify the relationships between MC concentration, cyanobacteria abundance, and environmental conditions in the hinterland of the TGRA. Our study offers novel insights into the effects of seasonal floods on MC concentration, cyanobacteria abundance, and environmental conditions in the hinterland of the TGRA, which could facilitate better management and conservation of this crucial freshwater resource.

## 2 Materials and methods

### 2.1 Study area and sampling sites

The study area was located in the Wanzhou district of Chongqing City, which is situated within the hinterland of the TGRA of the Yangtze River. It spans from 30°46′0″N, 108°24′0″E to 30°48′0″N, 108°21′0″E (Figure 1A). The range of water level elevation in Wanzhou District varies from 175 to 145 m (Tan and Schultz, 2021). Tan and Schultz (2021) demonstrated that Wanzhou district was greatly affected by these extreme seasonal floods. As depicted in Figure 1A, 12 representative sampling sites were selected in Wanzhou District, encompassing different geographical locations and hydrological conditions. These sampling sites were labeled as No. 1–12. Sites No. 1–9 were situated along the trunk stream of the Yangtze River, whereas sites No. 10–12 were located within the tributary stream of the Yangtze River, known as Zhuxi River. These sampling sites were selected to represent various cyanobacteria habitat types within the research area.

### 2.2 Sampling and pretreatment

We conducted monthly sampling of each site from July 2020 to October 2021. The sampling date was around the 20th of each month. Occasionally, due to weather conditions, the sampling dates for the 16-month period may vary, ranging from the 19 to the 24th of each month. A speed boat was rented to conduct sampling in the research area. Surface water samples (0.5 m depth) were collected by using a bob-weight grab sampler and were stored by a 5-L plastic bottle at each sampling site. Some water quality objectives, such as pH, dissolved oxygen (DO), and turbidity, were measured by using a portable multiparameter pH meter (Pro2Go, Mettler Toledo Co. Ltd., Zurich, Switzerland), a DO instrument (HQ40d, HACH Co. Ltd., Loveland, CO, United States), and a turbidimeter (2100Q, HACH Co. Ltd., Loveland, CO, United States) on the sampling sites, respectively. Conductivity and temperature were measured by a portable conductivity meter (HQ30d, HACH Co. Ltd., Loveland, CO, United States). Water samples were filtered through a 500-mesh steel sieve to remove suspended solids from the water samples preliminarily. Meanwhile, photos were taken to record visible changes in water quality during the flood (August 2020) and non-flood periods (August 2021) (Figure 1B). The settled water samples were digested for the analysis of total nitrogen (TN) and total phosphorus (TP) immediately after sampling (Liu et al., 2020; Bai et al., 2022; de Anda et al., 2022; Zhong et al., 2022; Zhen-Zhen et al., 2023). Subsequently, testing is performed after digestion. A total of 200 mL of the settled water samples was filtered through a 0.45 μm membrane filter, and the filtrate was stored at 4°C in brown glass bottles for the analysis of total dissolved phosphorus (TDP). We also filtered 1,500 mL of the settled water samples through the 0.22 μm organic membrane filters and stored the filtrates at 4°C in brown glass bottles to protect them from light. The 0.22 μm organic membrane filters were stored in sterile bottles at −80°C for DNA extraction. Moreover, the filtrates were used to measure of MC concentration.

### 2.3 Analysis of water quality objectives

TN, TP, and TDP were analyzed following the National Standard method (HJ636-2012, GB11893-89) with UV–visible spectrophotometer (DR6000, HACH Co. Ltd., Loveland, CO, United States). The settled water samples were digested by heating with alkaline potassium persulfate and added hydrochloric acid. Subsequently, we performed the concentration measure of TN in the processed water samples by comparing calibration curves (*R*^2^ = 0.9992). The settled water samples were digested by heating with potassium persulfate and adding 10% ascorbic acid solution and molybdate solution to coloration. Next, we performed the concentration measurement of TP in the processed water samples by comparing calibration curves (*R*^2^ = 0.9991). TDP was determined the same way (Liu et al., 2020; Bai et al., 2022; de Anda et al., 2022; Zhong et al., 2022; Zhen-Zhen et al., 2023). All chemical reagents in this study were applied with guaranteed reagent (Merck Co. Ltd., Darmstadt Germany).

### 2.4 Concentration measurement of microcystins

Microcystins were analyzed following the National Standard method (GB/T 20466-2006) with high-performance liquid chromatograph (HPLC; LC-2800G, Shimadzu Co. Ltd., Kyoto, Japan). The 1,000 mL of filtrate was enriched in a solid-phase extraction device through 1 g of C_18_ column (Waters Co. Ltd., Milford, Mass, United States). The C_18_ column was then leached and eluted by leaching solution and acidified methanol. The eluting solution was detected in HPLC after condensation and filtration. Three typical MCs (e.g., MCLR, MCYR, and MCRR) were detected in this study. We performed the concentration measure of MCs in condensed eluting solutions by comparing the calibration curves (MCLR, *R*^2^ = 0.9999; MCYR, *R*^2^ = 0.9999; and MCRR, *R*^2^ = 0.9998) (Martin et al., 2020, 2021; Hill et al., 2022; Painefilú et al., 2022).

### 2.5 DNA extraction and Illumina MiSeq sequencing

We selected two to four representative sample sites, including trunk stream and tributary stream, in each month after seasonal floods to perform DNA extraction. The sampling period of DNA extraction was from October 2020 to September 2021. In the present study, the number of DNA extraction samples was 35. Notably, the cryopreserved 0.22 μm organic membrane filters were used to perform DNA extraction.

Total DNA was extracted from 35 samples (35 for each treatment) using the E.Z.N.A.® soil DNA Kit (Omega Bio-tek, Norcross, GA, United States) according to the manufacturer’s protocol. All DNA samples were quality checked, and their concentrations were quantified by NanoDrop 2000 spectrophotometers (Thermo Fisher Scientific, Wilmington, DE, United States). Bacterial 16S rRNA gene fragments (V3-V4) were amplified from the extracted DNA using primers 338F (5′-ACTCCTACGGGAGGCAGCAG-3′) and 806R (5′-GGACTACHVGGGTWTCTAAT-3′) and the following PCR conditions: 30 s at 95°C, 30 s at 55°C, and 45 s at 72°C for 27 cycles. PCRs were performed with 4 μL 5 × TransStart FastPfu buffer, 2 μL 2.5 mM deoxynucleoside triphosphates (dNTPs), 0.8 μL of each primer (5 μM), 0.4 μL TransStart FastPfu DNA Polymerase, 10 ng of extracted DNA, and finally using ddH_2_O to make up 20 μL. Agarose gel electrophoresis was performed to verify the size of amplicons. Amplicons were subjected to paired-end sequencing on the Illumina MiSeq sequencing platform using PE300 chemical at Majorbio Bio-Pharm Technology Co. Ltd. (Shanghai, China).

### 2.6 Amplicon sequence processing and analysis

After demultiplexing, the resulting sequences were merged with FLASH (v1.2.11) (Magoč and Salzberg, 2011) and quality filtered with fastq (0.19.6) (Chen et al., 2018). Next, the high-quality sequences were de-noised using DADA2 plug in in the Qiime2 (version 2020.2) pipeline with recommended parameters, which obtains single-nucleotide resolution based on error profiles within samples (Callahan et al., 2016; Amir et al., 2017; Bolyen et al., 2019). DADA2 denoised sequences are usually called amplicon sequence variants (ASVs). To minimize the effects of sequencing depth on alpha and beta diversity measures, the number of sequences from each sample was rarefied to 4,000, which still yielded an average coverage of 97.90%. Taxonomic assignment of ASVs was performed using the Naive bayes consensus taxonomy classifier implemented in Qiime2 and the SILVA 16S rRNA database (v138). The 16S rRNA microbiome sequencing data were analyzed using the free online platform of Majorbio Cloud Platform.^1^ All sequencing data are available through the National Center for Biotechnology Information (NCBI) Sequence Read Archive under the accession number PRJNA1038780 (reference SRP 471467).

### 2.7 Data processing and analysis

We used Kruskal-Wallis H test followed by Welch’s *post hoc* test to compare the differences in cyanobacterial abundance and diversity among regions and months. Meanwhile, we performed Permutational Multivariate ANOVA (PERMANOVA) followed by pairwise tests to analyze the explanatory power of different environmental factors on sample differences. We used redundancy analysis to explore the relationships between cyanobacterial community composition and environmental variables. We used linear regression to examine the relationships between MC concentration and cyanobacterial abundance. We considered *p* < 0.05 as statistically significant in this research.

## 3 Results

### 3.1 Water quality objectives

**Figure 2:**
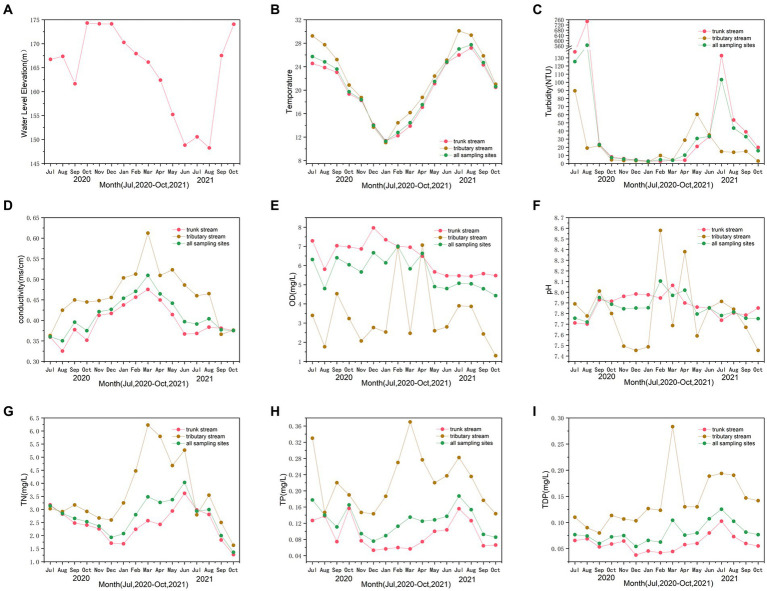
The water level elevation during the sampling period **(A)**. The trends of relevant water quality objectives of temperature **(B)**, turbidity **(C)**, conductivity **(D)**, dissolved oxygen (DO) **(E)**, pH **(F)**, total nitrogen (TN) **(G)**, total phosphorus (TP) **(H)**, and total dissolved phosphorus (TDP) **(I)** during the sampling period.

The water quality objectives in the hinterland of the TGRA are shown in Figure 2. Figure 2A represented the water level elevation of TGRA, whereas Figures 2B–I indicated the mean values of water quality objective concentration of sampling sites in trunk stream, tributary stream, and all sampling sites in the TGRA.

Figure 2A showed the variation in the water level elevation in the hinterland of TGRA. The water level elevation of TGRA remained at approximately 175 m during the storage period and began to decline in April to withstand possible seasonal floods. The water level elevation ranged from 148.25 to 174.30 m. The water level elevation during the flood period (July and August 2020) was 166.73 and 167.35 m. The water level elevation of the non-flood period (July and August 2021) was 150.55 and 148.25 m. Figure 2B displays the variation in water temperature throughout the sampling period. The water temperature ranged from 11.1°C in January 2021 to 30.1°C in July 2021. The line graph revealed fluctuations in variable turbidity, with periods of increase and decrease (Figure 2C). Turbidity ranged from 1.9 to 747.6 NTU. The variation in turbidity was from 2.7 to 7.2 NTU in winter and spring and from 10.2 to 103.2 NTU in summer and autumn of 2021. Moreover, the turbidity in flood period was from 23.1 to 564.4 NTU. The turbidity in flood period was significantly higher than that in the non-flood period (*p* = 0.001). Figure 2D shows that conductivity ranges from 0.325 to 0.612 ms·cm^−1^. The line graph demonstrated a volatile upward trend before March 2021 and a downward trend after that period. Frequent fluctuations in the DO values are shown from the line graph in Figure 2E. The DO ranged from 1.30 to 7.97 mg·L^−1^ and was higher in winter and spring and lower in summer and autumn for all sampling sites. This trend was more evident for the trunk stream. The line graph demonstrated volatility in pH, with frequent ups and downs (Figure 2F). The pH ranged from 7.45 to 8.58, with an average of 7.86. Figure 2F exhibited a gradual upward trend before February 2021 and a gradual downward trend after that.

As shown in Figure 2G, the line graph depicted substantial fluctuations in the mean values of TN concentration, with alternating peaks and valleys. The mean values ranged from 1.27 to 6.23 mg·L^−1^. The line graph displayed a gradual decreasing trend from July 2020 to December 2020. Subsequently, it started to rise until June 2021 and then showed decreasing trend in October 2021 for all sampling sites. Similar trend in TP can be seen by observing Figure 2H. It displayed that the range of the mean values of TP concentration was from 0.06 to 0.37 mg·L^−1^. The trend of mean values was decreasing in winter and spring and increasing in summer and autumn. Figure 2I also showed a similar trend in TDP. However, the mean TDP concentration values exhibited a steadier fluctuation than TP for all sampling sites, indicating a stable pattern.

For the entire study period, the mean values of water quality objectives for sampling sites in trunk and tributary stream exhibited a similar trend in all sampling sites. However, wider range fluctuation for all water quality objectives was observed in the tributary stream than in the trunk stream.

### 3.2 Microcystins

**Figure 3:**
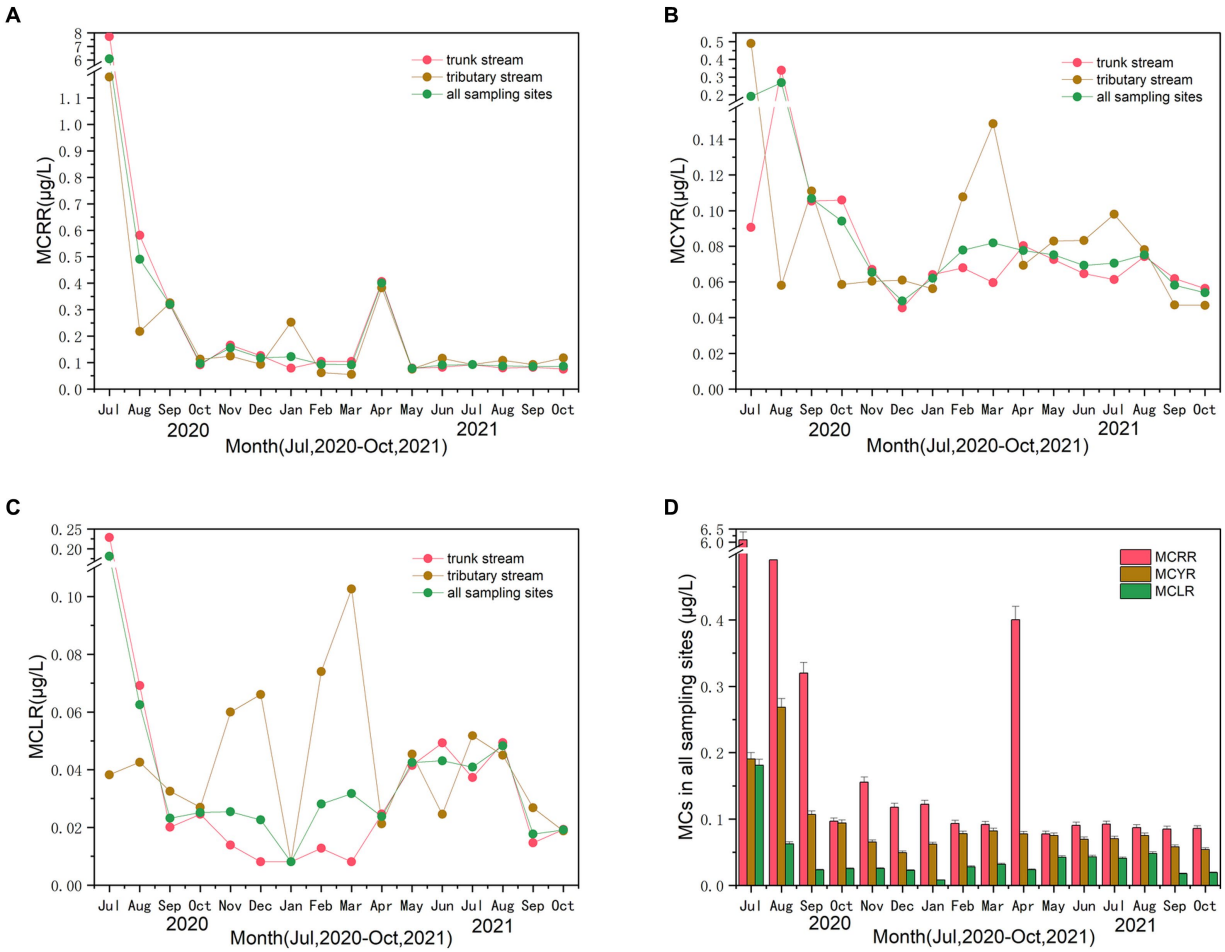
The trends of the three microcystin (MC) concentration of MCRR **(A)**, MCYR **(B)**, and MCLR **(C)** in trunk and tributary stream for the sampling period and all of sampling sites. The mean values of three MC concentrations in all sampling sites over the sampling period **(D)**.

Figure 3 depicts the fluctuating trends and the corresponding mean values of the three most common MC concentration (i.e., MCRR, MCYR, and MCLR) in trunk and tributary stream for the sampling period and all of sampling sites, with alternating periods of rise and fall. According to Figure 3A, a surge in the mean values of MCRR concentration was pronounced during the flood period (July and August 2020) and April 2021. The mean values ranged from 0.06 to 7.72 μg·L^−1^. Figure 3B shows the mean values of MCYR concentration. The mean values ranged from 0.05 to 0.49 μg·L^−1^. A significant increase was observed during the flood period and then decreased to approximately 0.05 μg·L^−1^ in December 2020. Thereafter, the mean values of MCYR concentration began to increase gradually in summer for all sampling sites. A downward trend occurred after August 2021. Figure 3C displays the mean values of MCLR concentration. The mean values ranged from 0.01 to 0.23 μg·L^−1^. The mean values of MCLR concentration increased during the flood period and then decreased to 0.01 μg·L^−1^ in January 2021. The MCLR concentration began to steadily rise to 0.05 μg·L^−1^ in August 2021. Subsequently, the concentration showed a downward trend after that for all sampling sites. Notably, Figures 3A–C illustrated significant increase during the flood period (July and August 2020) for MCRR (*p* = 0.011), MCYR (*p* = 0.048), and MCLR (*p* = 0.027) compared with the non-flood period (July and August 2021). Furthermore, the trend of concentration in trunk stream was similar to that in all sampling sites for MCRR, MCYR, and MCLR over the sampling period. Figure 3D depicts the mean values of three MC concentrations in all sampling sites over the sampling period. Based on the graph, the mean values of MCRR concentration were the highest among the three MCs, and the second highest MC concentration was achieved by MCYR. The mean values of MCLR concentration were the lowest among the MCs.

### 3.3 Species diversity and cyanobacteria abundance

**Figure 4:**
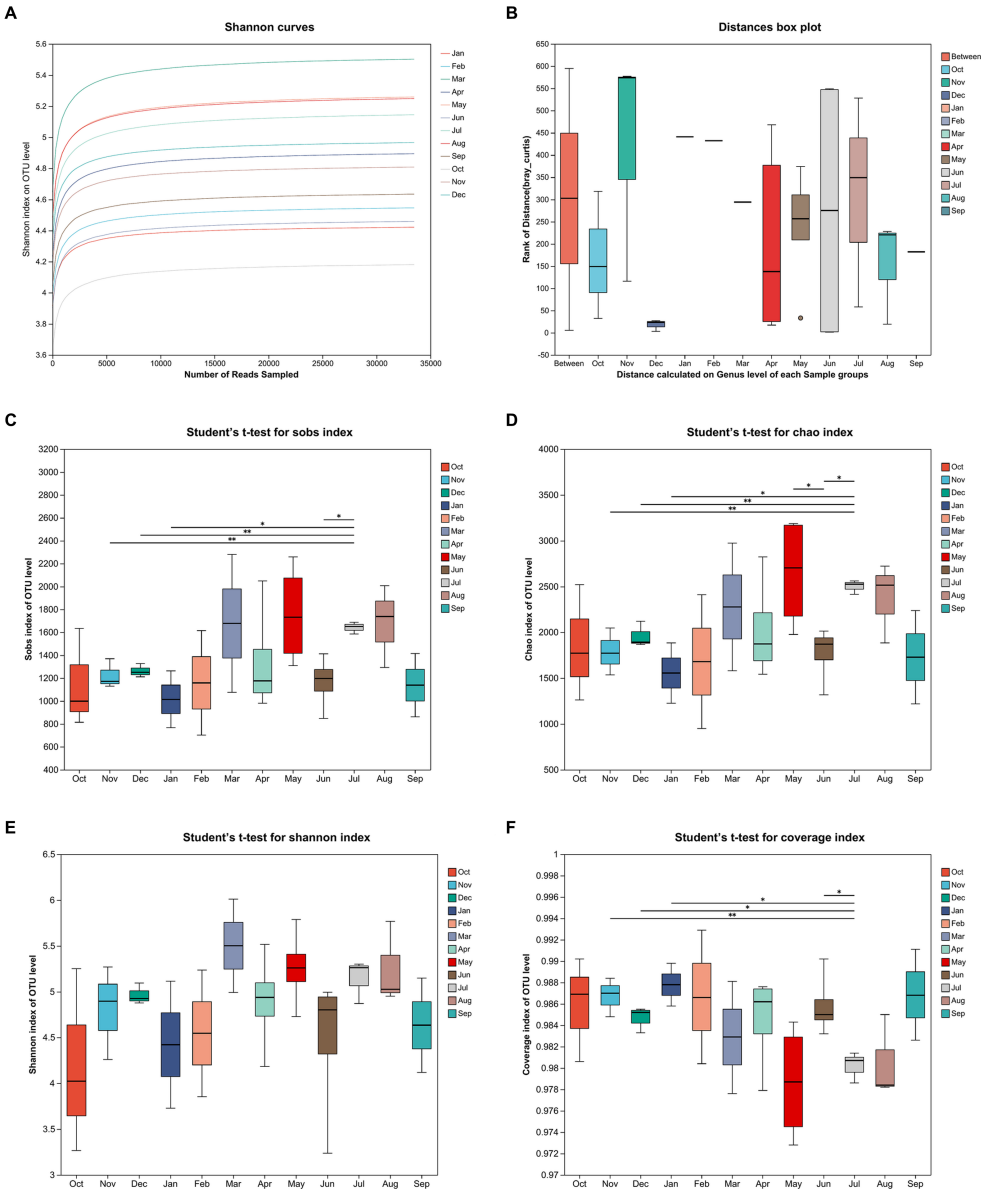
The Shannon curves on OTU level for sampling period **(A)**. Analysis of similarities (ANOSIM) of 12 sampling groups **(B)**. The mean values of sobs index **(C)**, chao index **(D)**, Shannon index **(E)**, and coverage index **(F)** of OTU level for sampling period.

The comparison and analysis of all species diversity results were based on 16S rRNA microbiome sequencing data obtained from monthly sampling that lasted for 12 months (October 2020 to September 2021) after the floods. And then all 35 samples are grouped by month. The Shannon curves on OTU level showed clear asymptotes (Figure 4A), which indicated a near-complete sampling of the community. Analysis of similarities (ANOSIM) was used to analyze the differences of distance between different groups and that within group samples (Figure 4B). The graph illustrated remarkable differences among the months by comparing the distance calculated on the genus level of each sample groups (Bray_Curtis ANOSIM, *r* = 0.21, *p* = 0.003). (The observed richness) Sobs index, Chao index, and Shannon index of OTU level showed similar tendencies, which showed an increase in winter and spring and a decrease during summer and autumn (Figures 4C–E). Moreover, Sobs index and Chao index of OTU level were significantly higher in July 2021 than in November–December 2020 and January 2021. Figure 4F indicates that the coverage index of OTU level in all of groups is over 0.97.

**Figure 5:**
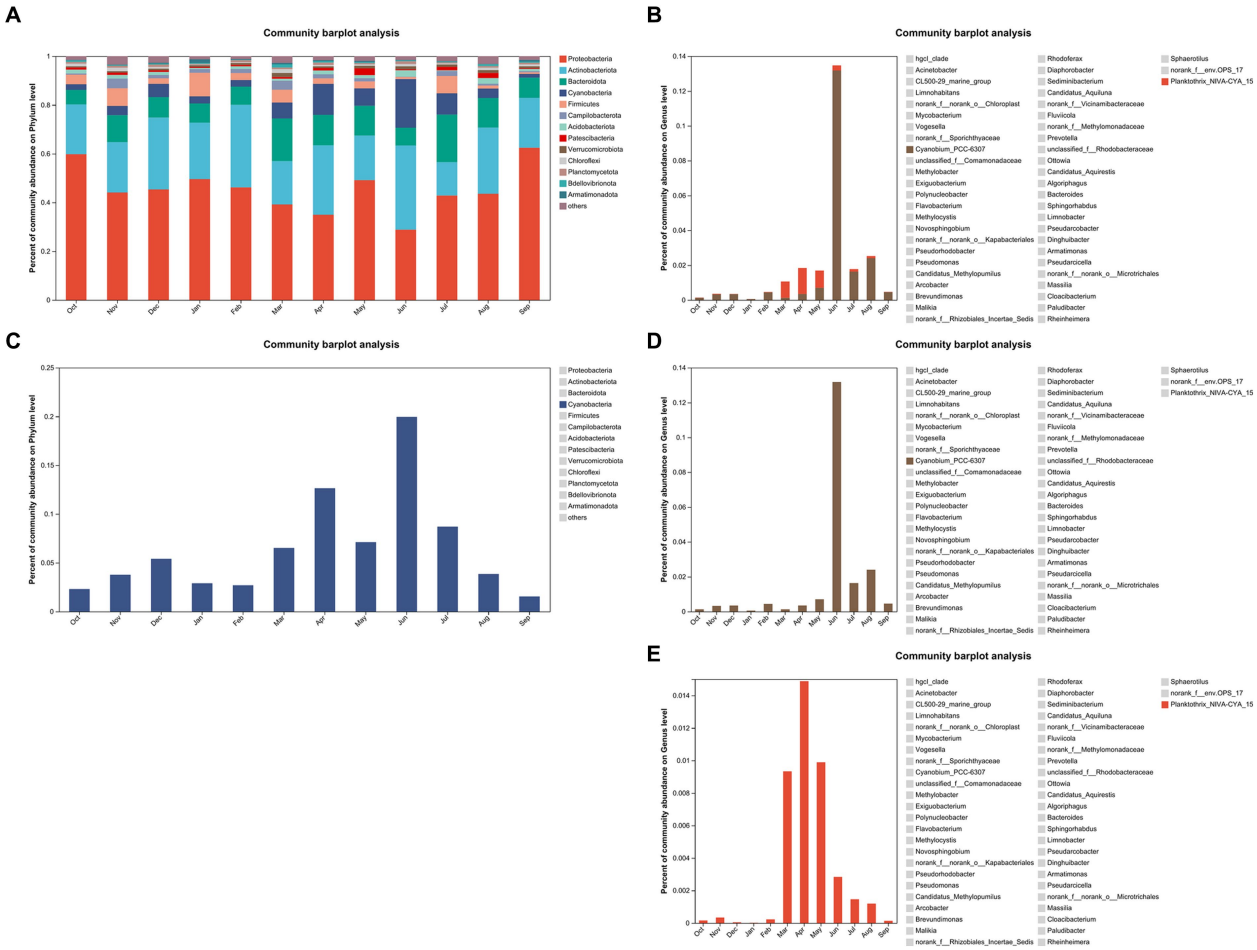
The proportion of community abundance **(A)** and cyanobacteria abundance **(C)** on the phylum level for all samples. The proportion of *Cyanobium_PCC-6307* and *Planktothrix_NIVA-CYA_15* abundance **(B)** on the genus level and the proportion of *Cyanobium_PCC-6307* abundance **(D)** and *Planktothrix_NIVA-CYA_15* abundance **(E)** on the genus level, respectively.

Figure 5A displays the proportion of the mean values of community abundance on the phylum level over the sampling period. Note that *Cyanobacteria* ranked fourth. The proportion of *Cyanobacteria* was displayed separately in Figure 5C. The percentage range of *Cyanobacteria* was from 1.55 to 19.95%. The graph demonstrated that *Cyanobacteria* abundance on phylum level was significantly higher in summer and autumn than it in spring and winter (*p* = 0.021). Furthermore, Figure 5C shows that the *Cyanobacteria* abundances on phylum level are higher in April and June 2021 than those in other months. Figure 5B displays the proportion of community abundance on the genus level over the sampling period. Two genera, namely, *Cyanobium_PCC-6307* and *Planktothrix_NIVA-CYA_15*, are included in *Cyanobacteria* phylum. The abundance of *Planktothrix_NIVA-CYA_15* was higher than that of *Cyanobium_PCC-6307* in March, April, and May 2021. Furthermore, the abundance of *Cyanobium_PCC-6307* was higher than that of *Planktothrix_NIVA-CYA_15* in other months. The abundances of *Cyanobium_PCC-6307* were significantly higher in June, July, and August 2021 than those in other months (*p* = 0.046, Figure 5D). The abundance of *Planktothrix_NIVA-CYA_15* was significantly higher from March to August 2021 than that in other months (*p* = 0.042, Figure 5E). Notably, the abundances of *Cyanobium_PCC-6307* and *Planktothrix_NIVA-CYA_15* exhibited the highest values in June and April 2021, respectively.

### 3.4 Analysis of sample comparison and species difference

**Figure 6:**
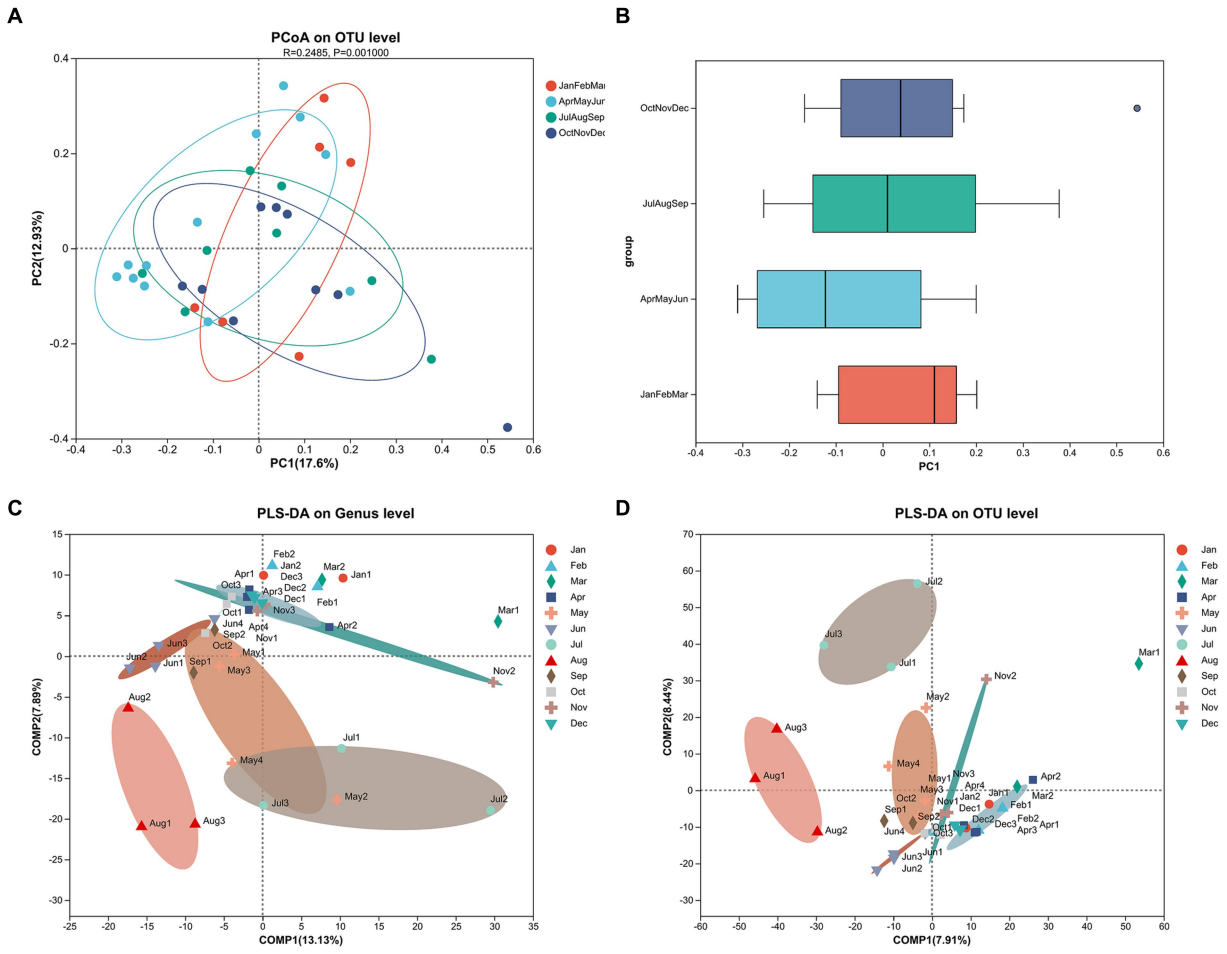
The principal coordinate analysis (PCoA) on OTU level by season **(A)** and corresponding discrete distribution by season **(B)**. The partial least square discriminant analysis (PLS-DA) on genus level **(C)** and OTU level **(D)** by month.

Figures 6A,B display principal coordinate analysis (PCoA) on OTU level by using the bray Curtis distance algorithm subgrouping by season. Figure 6A indicates the significant differences between seasons (PERMANOVA, *R*^2^ = 0.2485, *p* = 0.001). Figures 6C,D display the partial least square discriminant analysis (PLS-DA) on genus and OTU levels to analyze the difference of species composition between the sampling groups. Notably, Figure 6C indicates that the species composition on the genus level in May, June, July, and August had differences compared with other months.

**Figure 7:**
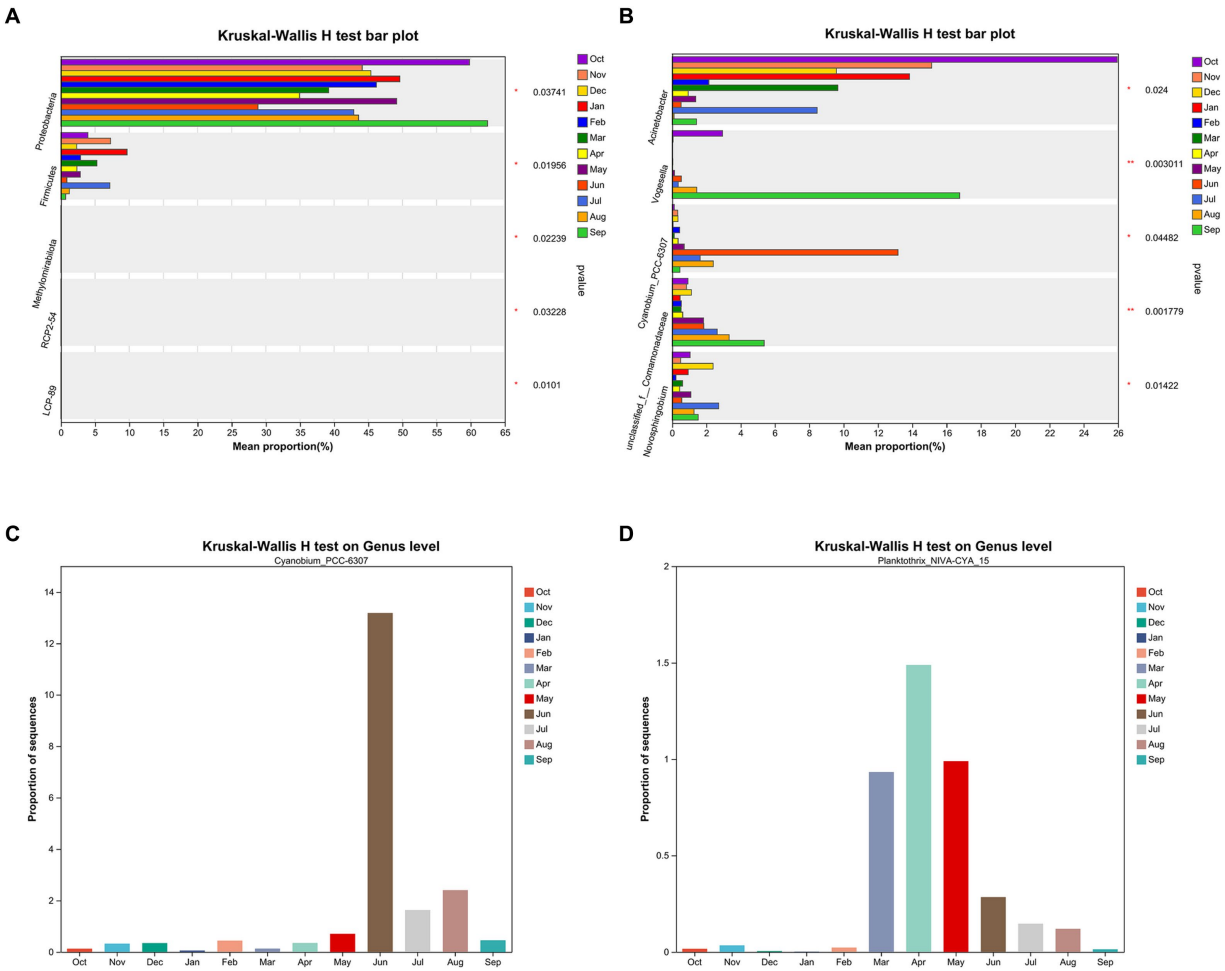
The Kruskal–Wallis H test top 5 species with significant differences on phylum level **(A)** and genus level **(B)** for all samples, respectively. The Kruskal–Wallis H test in *Cyanobium_PCC-6307* abundance **(C)** and *Planktothrix_NIVA-CYA_15* abundance **(D)** on genus level by month, respectively.

Figures 7A,B display the Kruskal–Wallis H test top 5 species with significant differences on phylum and genus levels for all samples, respectively. Figure 7B illustrates that *Cyanobium_PCC-6307* has significant differences on genus level in different months (Welch’s (uncorrected), *p* = 0.045). Figures 7C,D show the Kruskal–Wallis H test in *Cyanobium_PCC-6307* and *Planktothrix_NIVA-CYA_15*. Significant differences in summer and autumn were observed for *Planktothrix_NIVA-CYA_15* compared with winter and spring (*p* = 0.042).

### 3.5 Correlation analysis of environmental factors

The explanatory power of different environmental factors on sample differences were analyzed by PERMANOVA, and statistical significance analysis was conducted. Table 1 displays PERMANOVA by Jaccard distance algorithm on genus level for sampling environment factors. The *R*^2^ values represented the explanatory power of each factor to the sample differences, and a larger *R*^2^ value indicated a higher explanatory power of the environment factors to the sample differences. Therefore, the rank of explanation of sample differences was TN, TDP, OD, TP, conductivity, temperature, turbidity, and pH. Note that *p* values of TN, TDP, OD, TP, conductivity, temperature, and turbidity indicated that these environmental factors had reliable explanation for species differences on genus level (*p* < 0.05).

**Table 1:** Permutational Multivariate ANOVA (PERMANOVA) by Jaccard distance algorithm on genus level for sampling environment factors.

Characteristics (*n* = 35)	Sums of Sqs (*n* = 35)	Mean Sqs (*n* = 35)	F_Model (*n* = 35)	*R* ^2^	*p* value
TN	0.75309	0.75309	3.04033	0.08436	0.001
TDP	0.72780	0.72780	2.92916	0.08153	0.001
OD	0.68550	0.68550	2.74476	0.07679	0.001
TP	0.58032	0.58032	2.29432	0.06501	0.002
Conductivity	0.61978	0.61978	2.46197	0.06943	0.002
T	0.67929	0.67929	2.71783	0.07609	0.003
Turbidity	0.43145	0.43145	1.67585	0.04833	0.026
pH	0.35858	0.35858	1.38099	0.04017	0.098

## 4 Discussion

This study investigated the effects and seasonal variations in 2020 seasonal floods based on MC concentration, cyanobacteria abundance, and water quality objectives in the hinterland of the TGRA. Seasonal flood period (August 2020) was compared with non-flood period (August 2021) and suggested that the water quality significantly became more turbid during flood period than it was in non-flood period (Figure 1B). Based on the changes in water level elevation and temperature (Figures 2A,B), the TGRA exhibited the following characteristics: less water volume and higher temperatures during summer and autumn, and larger water volume and lower temperatures during spring and winter. Moreover, the water level elevation of the flood period (July and August 2020) was significantly higher than that in the non-flood period (July and August 2021) (*p* = 0.004). To summarize the findings, turbidity was significantly increased in the flood period. However, TN, TP, and TDP did not decrease despite the rise in water level elevation caused by the floods. This phenomenon might be attributed to increased surface runoff caused by floods, leading to water eutrophication. That is consistent with the previous study conducted by Bredin et al. (2020). Meanwhile, TN, TP, TDP, and turbidity showed the tendency of increasing in summer and autumn and decreasing in winter and spring (Figures 2G–I). TN, TP, and TDP tend to decrease in lower temperature and larger water volume and increase in higher temperature and less water volume. These findings might be attributed to the influence of the variation in temperature and water volume. The results are consistent with those in previous studies in water quality objectives (Liu et al., 2020; Bai et al., 2022; de Anda et al., 2022; Zhong et al., 2022; Zhen-Zhen et al., 2023). These results indicate that environmental conditions could affect cyanobacteria abundance.

Our MC concentration results indicate that the mean values of MCLR, MCYR, and MCRR concentration tend to increase significantly during flood period compared with the non-flood period. Meanwhile, the mean values of MCLR and MCYR have similar tendency with that in some water quality objectives (e.g., TN, TP, and TDP), that is, increasing in summer and autumn and decreasing in winter and spring (Figures 3A–C). These results are basically consistent with previous research in Lake Taihu and Lake Monona (Beversdorf et al., 2017; Xue et al., 2020). That may mean that water quality objectives would increase the concentration of MCs. However, the mean values of MCRR concentration only showed a significant increase in April 2021 after the occurrence of floods. This phenomenon indicates that MCRR concentration might be remarkably affected by cyanobacteria abundance in April 2021. Notably, the mean values of MCRR, MCYR, and MCLR concentration show significant increase during the flood period compared with the non-flood period (Figure 3D). The three MCs exhibited a trend where MCRR had the highest concnetration, followed by MCYR, and MCLR had the lowest concentration in the hinterland of TGRA. This phenomenon is consistent with the study of MCRR and MCLR by He et al. (2018) in the Yulin river. These results indicate that MCLR and MCRR might be common microcystin in the Yangtze River.

To investigate the reasons for the changes in MC concentration, we conducted monthly sampling for 12 months after floods and 16S rRNA microbiome sequencing to research the relationship between MC concentration and biological diversity, such as species diversity and cyanobacteria abundance. The flattened Shannon curves indicate that the sample data for the sampling period are sufficient (Figure 4A). ANOSIM indicated that the dissimilarity of species diversity on the genus level between sampling groups by month is significant (Figure 4B). Moreover, diversity index detection indicated that species diversity in summer and autumn is significantly higher than that in winter and spring (Figures 4C–E). The relationship of MC concentration and cyanobacteria abundance can be illustrated by showcasing species abundance (Figure 5). The tendency of cyanobacteria abundance on the phylum level is similar to those of MCLR and MCYR concentration (Figure 5C). The months with high *Cyanobium_PCC-6307* abundance on genus level (i.e., June, July, and August 2021) were consistent with that of MCLR (Figure 5D). Furthermore, the abundance of *Planktothrix_NIVA-CYA_15* on the genus level reached its highest level in April 2021, which is consistent with the tendency of MCRR (Figure 5E). Overall, these results indicate that the increase in cyanobacteria abundance might be an important factor that leads to the increase in MC concentration. Meanwhile, the increase in MCLR and MCRR concentration might be related to the increase in the abundance of *Cyanobium_PCC-6307* and *Planktothrix_NIVA-CYA_15*, respectively. These two cyanobacteria genera were also discovered in lakes, such as Lakes Erhai, Qionghai, and Xingyun in southwestern China and Yangtze River in Nanjing and Wuhan sections. That may mean these two cyanobacteria genera are commonly found in southern China. They are also closely related to the formation of MCs (Liu et al., 2022a,b; Bian et al., 2023).

The analysis of sample comparisons, as depicted in Figure 6, suggests significant differences in prokaryote composition at the OTU level among seasons. Notably, the months exhibiting differences in species composition at the genus level coincide with variations in MCLR concentrations (Figure 6C). This implies that differences in species composition may influence both cyanobacteria abundance and MC concentration. Furthermore, the analysis reveals significant seasonal variations in *Cyanobium_PCC-6307* and *Planktothrix_NIVA-CYA_15* through the assessment of species significance differences (Figure 7). These findings suggest that *Cyanobium_PCC-6307* and *Planktothrix_NIVA-CYA_15* are notably affected by seasonal factors. Moreover, the results of correlation analysis indicate a significant relationship between water quality parameters (i.e., TN, TDP, OD, TP, conductivity, temperature, and turbidity) and species variations (Table 1). Therefore, these findings imply a positive correlation between environmental conditions and cyanobacteria abundance. This correlation aligns with previous studies conducted in lakes and rivers, which have also confirmed this observation (Liu et al., 2022a,b; Bian et al., 2023).

This study has certain limitations. First, the spatial coverage of our sampling locations is limited, which may constrain the generalizability of our findings to a broader region. However, it is important to note that the Wanzhou section of the Yangtze River, serving as a representative area within the hinterland of the TGRA, holds significant importance for our research. To better understand the relationship between environmental conditions and MC concentration, future studies should consider sampling from various locations along the Yangtze River. Second, our study does not display instances where some water quality parameters and MC concentrations exceeded the established standards at specific sampling sites. Our primary focus in this study is to compare the variations in environmental conditions and MC concentration across different months to outline the trends during the study period. Consequently, we have presented the corresponding data for each month as mean values. In future research focused on spatial distribution, we intend to provide more comprehensive data. Lastly, the absence of historical data for comparison limits our ability to gain a more comprehensive understanding of long-term trends in cyanobacteria abundance. These historical data were not available for this study. Therefore, we plan to continue conducting sampling studies in different regions to further explore the relationship between MC concentration and environmental conditions.

## 5 Conclusion

In the current study, the 2020 seasonal floods led to a significant increase in the mean concentrations of MCRR, MCYR, MCLR, and certain water quality parameters (e.g., turbidity) in the hinterland of the TGRA when compared to non-flood periods. Interestingly, the concentrations of some other water quality parameters (namely, TN, TP, and TDP) remained similar during floods as they did during non-flood periods, despite a significant rise in water level due to the floods. This phenomenon can be attributed to the heightened surface runoff resulting from the floods. Furthermore, our findings indicate a clear trend of increasing mean concentrations for certain water quality parameters (TN, TP, TDP, and turbidity), MCs (MCRR, MCYR, and MCLR), and cyanobacteria abundance (*Cyanobium_PCC-6307* and *Planktothrix_NIVA-CYA_15*) during the summer and autumn seasons, with a subsequent decrease during the winter and spring in the hinterland of the TGRA over the course of 12 months following the floods. This pattern may be linked to rising temperatures that lead to an increase in the concentrations of water quality parameters, potentially resulting in water eutrophication. Consequently, water eutrophication can lead to an increase in cyanobacteria abundance and MC concentration. Additionally, the biodiversity in the hinterland of the TGRA also exhibits significant seasonal correlations. Notably, the concentrations of MCLR and MCRR show specific correlations with the abundance of *Cyanobium_PCC-6307* and *Planktothrix_NIVA-CYA-15*, respectively. For future studies, conducting additional sampling studies in different regions is essential to further investigate the relationship between MC concentration and environmental conditions.

## Data availability statement

The datasets presented in this study can be found in online repositories. The names of the repository/repositories and accession number(s) can be found in the article/supplementary material.

## Author contributions

YZ: Conceptualization, Data curation, Formal Analysis, Project administration, Visualization, Writing – original draft, Writing – review & editing. QW: Investigation, Methodology, Software, Writing – review & editing. GX: Resources, Validation, Writing – review & editing. ZZ: Funding acquisition, Supervision, Writing – review & editing.

## Footnotes

^1^ cloud.majorbio.com
